# The Risk of Age-Related Macular Degeneration Is Reduced in Type 2 Diabetes Patients Who Use Metformin

**DOI:** 10.3390/ph16020224

**Published:** 2023-02-01

**Authors:** Chin-Hsiao Tseng

**Affiliations:** 1 Department of Internal Medicine, National Taiwan University College of Medicine, Taipei 10051, Taiwan; ccktsh@ms6.hinet.net; 2 Division of Endocrinology and Metabolism, Department of Internal Medicine, National Taiwan University Hospital, Taipei 10002, Taiwan; 3 National Institute of Environmental Health Sciences of the National Health Research Institutes, Zhunan 35053, Taiwan

**Keywords:** age-related macular degeneration, metformin, pharmacoepidemiology, propensity score, Taiwan

## Abstract

Background: Whether metformin may reduce the risk of age-related macular degeneration (AMD) requires confirmation. This study compared the risk of AMD between ever users and never users of metformin matched on propensity score (PS) in Taiwanese patients with type 2 diabetes mellitus. Methods: We enrolled study subjects from Taiwan’s National Health Insurance. A total of 423,949 patients with new onset diabetes from 1999 to 2005 were identified. After excluding ineligible patients and enrolling only patients aged between 50 and 79 years, we created 13,303 pairs of ever users and never users of metformin matched on PS. The patients were followed from 1 January 2006 to 31 December 2011. We estimated hazard ratios by Cox regression. Results: AMD was newly diagnosed in 506 ever users and 639 never users. The respective incidence rates (per 100,000 person-years) were 778.72 and 1016.62. The hazard ratio (HR) and 95% confidence interval (CI) for ever versus never users was 0.756 (0.673–0.850). While ever users were categorized by tertiles of cumulative duration (<31.8, 31.8–63.9 and >63.9 months) and cumulative dose (<947.1, 947.1–2193.5 and >2193.5 g) of metformin, a dose–response pattern was observed. For the respective tertiles of cumulative duration, the HRs (95% CIs) were 1.131 (0.961–1.330), 0.821 (0.697–0.967) and 0.464 (0.384–0.561), while compared to never users. For the respective tertiles of cumulative dose, the HRs (95% CIs) were 1.131 (0.962–1.329), 0.739 (0.624–0.876) and 0.525 (0.438–0.629). A risk reduction among ever users was observed for all tertiles of defined daily dose but was most remarkable for the third tertile with a defined daily dose of >0.64. Subgroup analyses suggested that the benefit of metformin could be similarly observed among men and women and for age subgroups of 50–64 and 65–79 years. However, patients with diabetic retinopathy would not be significantly benefited and metformin did not seem to be preventive for exudative AMD. Conclusion: In general, metformin significantly reduces the risk of AMD.

## 1. Introduction

In elderly people, the major cause of blindness is age-related macular degeneration (AMD) [1]. AMD affects the macula of the retina in the eyes and is clinically progressive [1]. The etiology of AMD remains unclear but may involve genes and non-genetic risk factors such as smoking and low intake of antioxidants such as zinc and carotenoids [1]. Other major risk factors include obesity, history of cardiovascular/cerebrovascular disease, hypertension, diabetes, dyslipidemia, high plasma fibrinogen and a blue iris [2,3]. A meta-analysis has suggested that age, smoking, cataract surgery and family history of AMD are strongly associated with late AMD [2].

Clinically, AMD can be classified as early stage, with drusen and abnormalities of the retinal pigment epithelium, or late stage, characterized by neovascularization (wet or exudative) or atrophy (non-neovascular, dry or non-exudative) [3]. Antioxidants can be used to slow the progression from early to late stage and anti-vascular endothelial growth factor (anti-VEGF) therapy may show some effects in the treatment of neovascularization [3]. There are no proven therapies for AMD with atrophy [3]. The clinical course of AMD is characterized by progressive loss of central visual acuity leading to visual impairment and finally blindness [3]. It was estimated that there were 200 million cases of AMD in 2020 and the case number will increase to 300 million by 2040 [4].

Metformin is an activator of the 5′-adenosine monophosphate-activated protein kinase (AMPK). It is currently the first-line antidiabetic drug used to treat hyperglycemia in patients with type 2 diabetes mellitus (T2DM) [5]. Metformin exerts a glucose-lowering effect with additional multiple pleiotropic benefits including anti-atherosclerosis, anti-inflammation, anti-neoplasm, anti-aging, anti-microbial, pro-osteogenesis and immune modulation [6,7,8,9,10,11,12,13,14,15,16,17].

Metformin crosses the blood–brain barrier (BBB) and can be detected in various regions of the brain including the hypothalamus, pituitary gland and frontal cortex [18,19]. In Wistar rats, metformin concentration peaks in the brain 6 h after oral administration and a high BBB penetrance can be demonstrated by the high brain to plasma ratio of 0.99 [20]. An animal study showed that metformin can cross the BBB and reach the retina, where it can stimulate AMPK and prevent degeneration of photoreceptors and the retinal pigment epithelium [21].

In humans, several recent observational studies suggested a protective effect of metformin on AMD [22,23,24,25,26]. However, a recently published paper that used a US insurance claims database showed conflicting association between metformin exposure and development of dry AMD [27]. While active users showed a significantly higher risk of 8%, prior users had a significantly lower risk of 5% [27]. A significant trend toward increased hazard with increasing cumulative dosage was observed in the cumulative dosage model. However, a significantly decreased risk (hazard ratio (HR): 0.95, 95% confidence interval (CI): 0.91–0.99) was seen in the lowest dosage quartile and a significantly higher risk (HR: 1.07, 95% CI: 1.01–1.13) in the highest quartile [27]. Therefore, the benefits of metformin on AMD require further clarification.

To further explore the risk of AMD with regard to metformin exposure in patients with T2DM, we used the nationwide reimbursement database of Taiwan’s National Health Insurance (NHI) to conduct a retrospective cohort study.

## 2. Results

The characteristics of never users and ever users in the propensity score (PS)-matched cohort and the standardized differences between the two groups are shown in Table 3. The values of standardized difference were <10% for all covariates, suggesting that the selected ever users and never users of metformin were well balanced in all covariates in the matched cohort.

**Figure 1:**
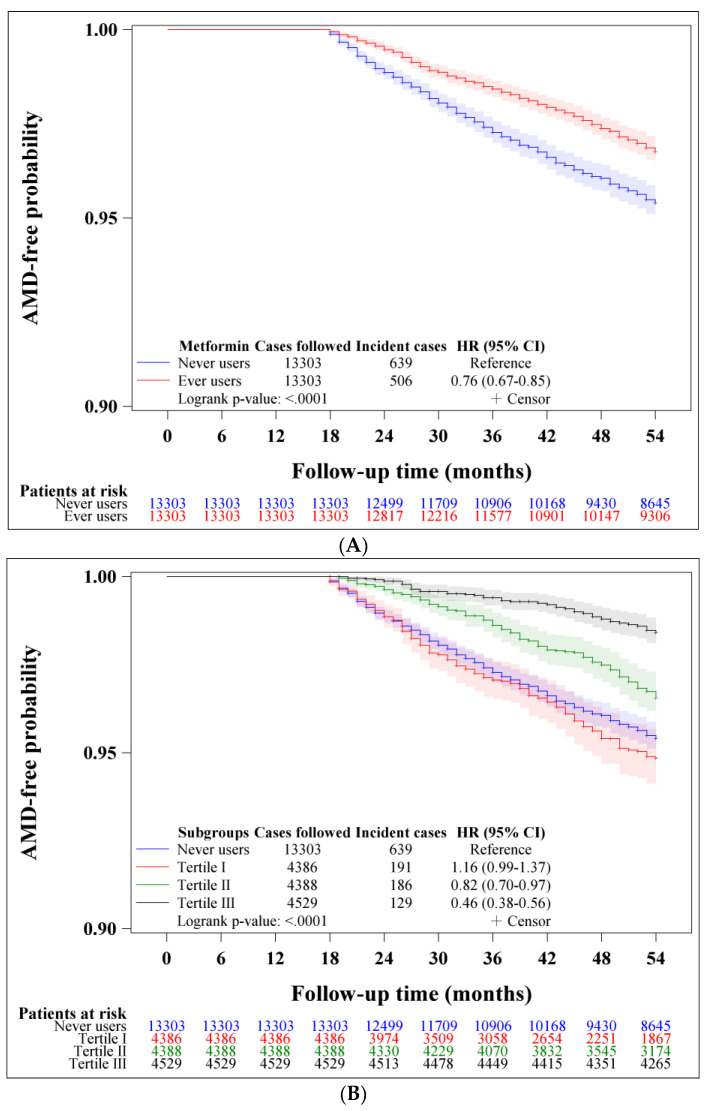

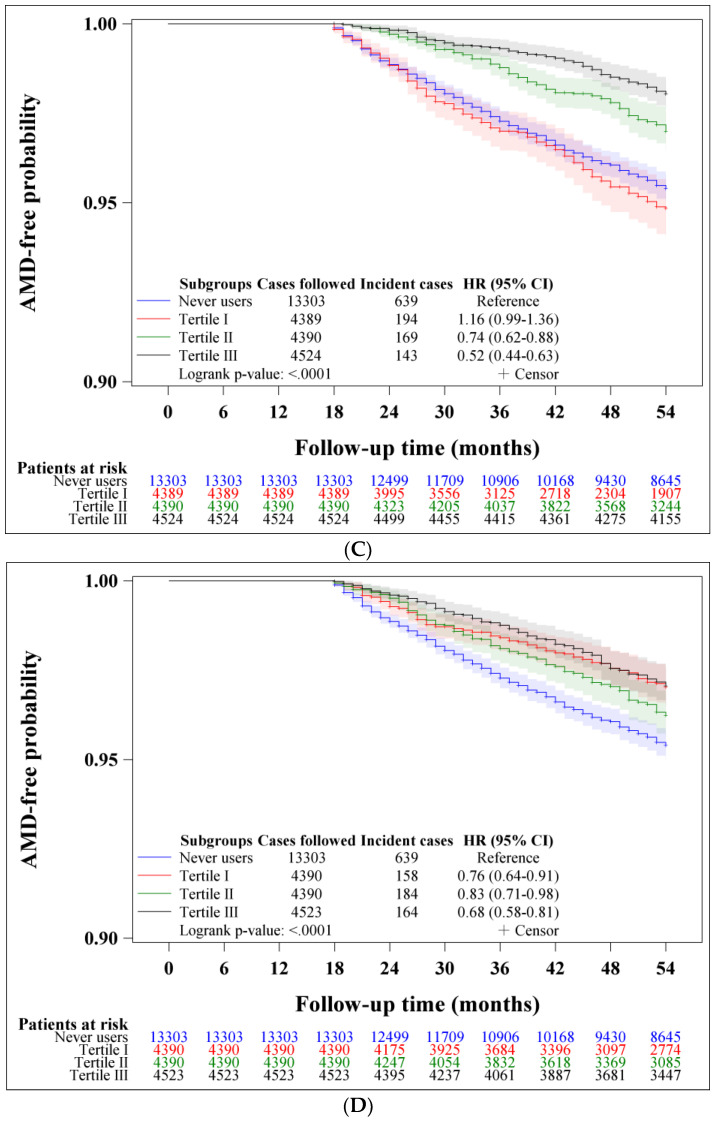
(**A**) Kaplan–Meier curves for age-related macular degeneration (AMD) in metformin never users and ever users. (**B**) Kaplan–Meier curves for age-related macular degeneration (AMD) in metformin never users and ever users categorized by the tertiles of cumulative duration. (**C**) Kaplan–Meier curves for age-related macular degeneration (AMD) in metformin never users and ever users categorized by the tertiles of cumulative dose. (**D**) Kaplan–Meier curves for age-related macular degeneration (AMD) in metformin never users and ever users categorized by the tertiles of defined daily dose of metformin.

Figure 1 shows the Kaplan–Meier curves for AMD with regard to metformin exposure, which indicated a lower risk associated with metformin use. The curves for ever users and never users are shown in Figure 1A (*p* < 0.0001, log-rank test). A significant dose–response relationship could be seen in the curves for never users and the tertiles of cumulative duration (Figure 1B) and cumulative dose (Figure 1C). A significant risk reduction could be seen in all tertiles of DDD (Figure 1D).

Table 1 shows the results of the main analyses on the incidence of AMD and the HRs comparing different subgroups of metformin exposure to never users of metformin. After a median follow-up of 5.57 years in never users and 5.60 years in ever users, the respective incidences were 1016.62 and 778.72 per 100,000 person-years. A significantly 24.4% lower risk was observed in ever users, as indicated by the overall HR that compared ever to never users. However, the proportional hazards assumption was violated in the estimation of this overall HR because the *p*-value of Schoenfeld’s global test was <0.05. The violation of the assumption might have implied a possibility of biased estimate of the HR. However, in the model that estimated the overall HR with additional adjustment for all covariates, the estimated HR was 0.759 (95% confidence interval 0.675–0.853, *p* < 0.0001) and the *p*-value of Schoenfeld’s global test in this model was >0.1. The unbiased estimate derived from this additional model did not markedly differ from the estimated HR of 0.756 (95% confidence interval 0.673–0.850) shown in Table 2, even though this unadjusted model did not meet the proportional hazards assumption.

The tertile analyses in Table 1 suggested a dose–response relationship in terms of metformin exposure indicated either by cumulative duration or cumulative dose. The tertile analysis on DDD suggested that the benefit could be observed in any of the DDD but the benefit would be greatest when the DDD was >0.64.

The sensitivity analyses restricted to various subgroups of patients are shown in Table 2. Except for the non-significant hazard ratios derived from the models that were conducted in patients with diabetic retinopathy (model 8) and for the outcome defined as exudative AMD (model 11), all other models consistently supported that metformin ever users would have a significantly lower risk of AMD. The significant risk reduction in ever users in comparison to never users could be seen in the age subgroups of 50–64 years (model 3) and 65–79 years (model 4). The preventive effect of metformin on AMD seemed to be similar in men (model 5) and in women (model 6).

## 3. Materials and Methods

Taiwan started to implement a nationwide and universal healthcare system, the NHI, on 1 March 1995. This healthcare system covers >99% of the Taiwan’s population. The insurants can receive medical care from all in-hospitals and >93% of all medical settings in Taiwan. Medical records submitted to the Bureau of the NHI for reimbursement have to be stored as computerized files. The database of these medical records includes disease diagnoses, drug prescriptions and clinical procedures. Researchers can submit research proposals for institutional review to request for the approval of the use of the database for academic research. The present study was approved by the Research Ethics Committee of the National Health Research Institutes (approval number 99274). As stipulated by local regulations, personal information must be de-identified before the release of the database. Therefore, informed consent was not required for the use of the database because there was no way to contact the patients.

Disease diagnoses were coded according to the International Classification of Diseases, Ninth Revision, Clinical Modification (ICD-9-CM) in the NHI database during the study period. Therefore, a diagnosis of diabetes mellitus was made by using the ICD-9-CM codes of 250.XX and the investigated outcome of AMD was defined by codes of 362.5X made by an ophthalmologist. The specific codes of AMD include 362.50 macular degeneration (senile), unspecified, 362.51 nonexudative senile macular degeneration, 362.52 exudative senile macular degeneration, 362.53 cystoid macular degeneration, 362.54 macular cyst, hole or pseudohole, 362.55 toxic maculopathy, 362.56 macular puckering and 362.57 drusen (degenerative).

**Figure 2:**
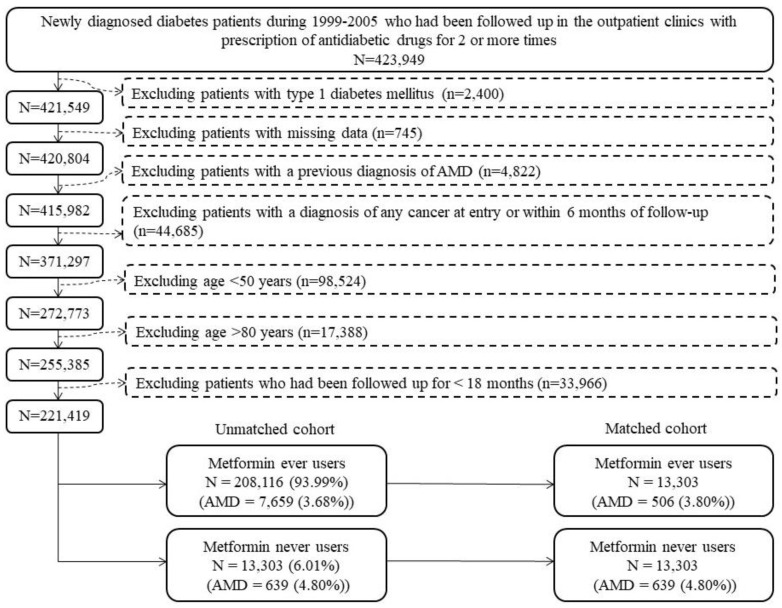
Procedures followed to create a propensity score-matched cohort of ever users and never users of metformin. AMD: age-related macular degeneration.

Figure 2 shows the procedures that we followed in creating a cohort of PS-matched ever users and never users of metformin. At first, we identified 423,949 patients who had been diagnosed with new-onset diabetes mellitus from 1999 to 2005. To ensure that the enrolled patients were newly diagnosed with diabetes mellitus during the enrollment period, we checked the database and excluded patients who had a diagnosis of diabetes mellitus during the period from 1995 to 1998. All patients should have received treatment with oral antidiabetic drugs and/or insulin for two or more prescriptions in the outpatient clinics to ascertain a diagnosis of diabetes mellitus. We then excluded ineligible patients according to the steps shown in Figure 2. As a result, 221,419 patients, 208,116 ever users and 13,303 never users of metformin, were enrolled as the unmatched cohort. PS was created by logistic regression from all variables in Table 3 plus the date of entry. A PS-matched cohort of 13,303 ever users and 13,303 never users, the matched cohort, was created based on the Greedy 8 → 1 digit match algorithm [15].

Patients aged <50 years were excluded because AMD is rare in the younger aged patients, and patients aged ≥80 years were excluded to avoid a potential bias resulting from healthy survivors. To examine whether there would really be a potential bias by including patients aged ≥80, we calculated the incidence of AMD stratified by age (<50, 50–54, 55–59, 60–64, 65–69, 70–74, 75–79, 80–84 and ≥80 years) and sex by including patients of all ages. We did observe a trend of increasing incidence in corresponding to increasing age in both men and women from <50 years of age up to the age group of 75–79 years and a decline in AMD incidence could be seen after the age of 80 years, suggesting a possible healthy survivor effect if patients older than 80 years were included (Supplementary Table S1).

Some potential confounders were retrieved from the database and they were listed in Table 3. The living regions of the patients were classified into the following five geographical locations: Taipei, Northern, Central, Southern and Kao-Ping/Eastern.

Occupation was categorized according to the Bureau of the NHI [17]: (I) civil servants, teachers, employees of governmental or private businesses, professionals and technicians; (II) people without a specific employer, self-employed people and seamen; (III) farmers and fishermen; and (IV) low-income families supported by social welfare and veterans.

The ICD-9-CM codes for most of the disease diagnoses had been previously reported [16,17]. Codes not reported in these previous papers are: hypoglycemia (251.0. 251.1 and 251.2), suicidal attempt (E950–E959), insomnia (780.52), diseases of the ear and mastoid process (380–388, excluding 389), hearing loss (389, 388.2), inflammatory diseases of the central nervous system (encephalitis and meningitis, 320–326), tuberculosis (010–018), malaria (084), some parasitic diseases (120–139), epilepsy and recurrent seizures (345), disorders of fluid electrolyte and acid-base balance (276) and cancer (140–208). Except for the variable of “cancer during follow-up” (patients with cancer at the start of follow-up had been excluded, Figure 2), all other variables were defined at the start of follow-up.

The accuracy of the ICD-9-CM codes in the database have been assessed by some other investigators [28,29]. In one study, the sensitivity and positive predictive value for a diagnosis of diabetes mellitus by using the ICD-9-CM codes of 250.XX were 90.9% and 90.2%, respectively [28]. In another study, Kappa values between claim data and medical records ranged from 0.55 to 0.86, suggesting moderate to substantial agreements [29].

Standardized difference was calculated for each variable [17,30]. We used a generally recommended cutoff value of standardized difference of >10% as an indicator of potential confounding [17,30].

We calculated cumulative duration and cumulative dose of metformin therapy from the database, and their tertiles were used to assess a dose–response relationship. As mentioned in a previously published paper [31], cumulative duration was calculated by accumulating the days of metformin prescriptions in all visits within the study period and was expressed in months by dividing the accumulated number of days by 30. Cumulative dose was calculated by summating the total doses of metformin in mg prescribed during the study period. Additionally, the defined daily dose (DDD) of metformin was used to investigate whether the risk might differ with regard to the daily dose of metformin [31]. One unit of DDD of metformin is equal to 2 g.

Incidence density was calculated in subgroups of never users, ever users and subgroups of ever users divided by the tertiles of cumulative duration, cumulative dose and DDD. Follow-up started on 1 January 2006. The incidence numerator was calculated as the case number of AMD newly diagnosed after follow-up. The incidence denominator, expressed in person-years, was the follow-up time calculated from the start of follow-up until a new diagnosis of AMD, the last available record in the reimbursement database or death, whichever occurred first, up to 31 December 2011.

Kaplan–Meier curves for AMD were plotted with regard to metformin exposure in the following subgroups: never users versus ever users and never users versus different tertiles of cumulative duration, cumulative dose and DDD. We used the log-rank test to examine the differences among different subgroups of metformin exposure.

We estimated HRs and their 95% CIs by using Cox regression. In the main analyses, we estimated the overall HR comparing ever users to never users and the HR comparing each tertile of cumulative duration, cumulative dose and DDD to never users. We used Schoenfeld’s global test to examine whether the estimate of the overall HR would violate the proportional hazards assumption of the model [32]. To examine whether the overall HR would be markedly different by using a different approach, we additionally estimated the HR for ever users versus never users by using a Cox proportional hazards regression that included all variables in Table 3 as independent variables. Similarly, Schoenfeld’s global test was used to examine whether this additional model would meet the requirement of the proportional hazards assumption.

Sensitivity analyses were conducted in more restricted subgroups to estimate the HRs for ever users versus never users:

1. Analysis restricted to patients enrolled during 1999–2002;
2. Analysis restricted to patients enrolled during 2003–2005;
3. Including only patients aged 50–64 years;
4. Including only patients aged 65–79 years;
5. Including only male patients;
6. Including only female patients;
7. Excluding patients with a diagnosis of anemia (ICD-9-CM 280–285) and/or nutritional deficiency (ICD-9-CM 260–269);
8. Patients with diabetic retinopathy (ICD-9-CM 362.0X);
9. Patients without diabetic retinopathy (ICD-9-CM 362.0X);
10. Outcome defined as nonexudative AMD (ICD-9-CM 362.50 and 362.51);
11. Outcome defined as exudative AMD (ICD-9-CM 362.52);
12. All covariates defined at censor.

Statistical analyses were conducted by using SAS statistical software version 9.4 (SAS Institute, Cary, NC, USA). We used a *p*-value cutoff of <0.05 as an indicator of statistical significance.

## 4. Discussion

### 4.1. Main Findings

The results of this study supported a lower risk of AMD in ever users of metformin when compared to never users (Table 2). A dose–response pattern with regard to metformin exposure in the tertile analyses of the cumulative duration and cumulative dose (Table 2) suggested a potential cause-effect relationship. The tertile analysis of DDD suggested that the benefit of metformin could be seen in any daily dose but patients who had a DDD of >0.64 showed the most remarkable risk reduction (Table 2). The sensitivity analyses suggested that the benefit of metformin could be demonstrated in any subgroups of age and sex (models 3–6, Table 2). However, patients who suffered from diabetic retinopathy (model 8, Table 2) would not be benefited from metformin treatment. The benefit of metformin seemed to be effective only for non-exudative AMD (model 10, Table 2) and not for exudative AMD (model 11, Table 2).

The conflicting association between metformin exposure and AMD reported by Eton et al. [27] was not similarly observed in this observational study conducted in the Taiwanese patients with T2DM.

### 4.2. Potential Mechanisms

Although not yet completely researched, the glucose lowering effect and the anti-aging, anti-inflammation, anti-oxidation and immune modulatory effects of metformin [6] might have contributed to such a reduced risk. Metformin may also influence the development of AMD by modifying gut microbiota.

Mitochondrial dysfunction can lead to chronic oxidative stress and is observed in patients with AMD [33]. A cellular study suggested that metformin protects retinal pigment epithelial cells from oxidative damage induced by hydrogen peroxide by enhancing autophagy through AMPK activation [34]. This finding was supported by another cellular and animal study that showed a protective effect of metformin on glyoxal-induced oxidative stress in retinal pigment epithelial cells [35]. AMPK regulates mitochondrial biogenesis through activating peroxisome proliferator-activated receptor gamma coactivator 1 and promotes mitochondrial fission by phosphorylation of mitochondrial fission factor [33]. AMPK activation by metformin may also phosphorylate and activate a protein involved in autophagy, a process that removes the damaged mitochondria [33,36]. Therefore, metformin may prevent the development of AMD either by promoting the biogenesis of new and healthy mitochondria or by removing damaged mitochondria [33].

Microbiota may play a significant role in eye diseases such as autoimmune uveitis, diabetic retinopathy and glaucoma [37]. However, there is a lack of study for such a potential link between AMD and gut microbiota [37]. Gut dysbiosis may cause systemic inflammation, metabolic disturbances and changes in metabolites that may signal distantly in the brain and the eye; therefore, it is possible that gut microbiota may also affect the development of AMD [37].

Metformin exerts an anti-aging effect [6]; therefore, it is possible that the delaying of the aging process also delays the development of AMD.

### 4.3. Clinical Implications

There are some clinical implications in this study. First, metformin’s preventive role in AMD provides an additional bonus beyond its glucose-lowering and other pleiotropic effects. AMD is very common in the aged population and significantly affects the quality of life and survival of the patients; therefore, clinical and economical burdens of AMD can be much reduced by a very inexpensive antidiabetic drug.

Second, because AMD is associated with cardiovascular disease [38,39,40,41,42,43,44], renal disease [38,45,46,47], periodontal disease [48,49,50,51,52,53] and Alzheimer’s disease [54,55,56], prevention of AMD may also reduce the burden of these common diseases. This would remarkably amplify the clinical significance following the prevention of AMD after metformin use.

Third, because there is a dose–response effect in the risk reduction of AMD associated with metformin use in terms of cumulative duration and cumulative dose (Table 2) and because the mechanisms might not simply be explained by glycemic control, it seems reasonable to maintain the use of metformin if the patients do not have any contraindication, even when other antidiabetic drugs are added for better improvement of hyperglycemia.

Fourth, the findings provide good rationale and references for the conduction of clinical trials to verify the beneficial effects of metformin on AMD. A phase 2 clinical trial is being conducted to investigate metformin’s effect on AMD [57,58] and a recent study suggested that the effect of anti-VEGF in the treatment of diabetic macular edema could be enhanced by metformin [59]. Some bio-nanotechnologies are being developed to improve the delivery of metformin and probucol as potential antioxidants to block the formation of reactive oxygen species for the treatment of chemotherapy-induced hearing loss [60] or age-related hearing loss [61]. Therefore, the use of metformin in combination with anti-VEGF in the treatment of AMD and the development of novel molecules with enhanced delivery of metformin to the retina are worthy of development.

Fifth, according to the results shown in Table 1, patients should have been treated for at least 31.8 months (second or third tertile of cumulative duration) or with a cumulative dose of 947.1 g (second or third tertile of cumulative dose) to demonstrate a significant risk reduction of AMD. Although patients who used a daily dose of 1 g (first tertile of DDD) might also have a reduced risk, it would take at least 2.6 years (947.1 g/365 days) to reach a cumulative dose of >947.1 g to demonstrate a significant risk reduction. Although patients who used a higher daily dose would reach a cumulative dose associated with a significant protection earlier, this should be balanced by the possible gastrointestinal side effects associated with a higher dose of metformin.

Sixth, because of the requirements of sufficient cumulative duration and cumulative dose for a significant risk reduction to be seen (Table 1) and because metformin’s benefit on AMD would only be significantly seen in patients without diabetic retinopathy (model 9, Table 2), metformin should be initiated earlier after diabetes diagnosis (especially before the development of diabetic retinopathy), increased to the maximal tolerable daily dose and continued (if not contraindicated) to get a better and earlier protective benefit.

Seventh, the lack of protection against exudative AMD (model 11, Table 2) supported that some unmodifiable risk factors such as age and family history of AMD may play important roles in the development of late AMD, as having been reported in a previous meta-analysis [2].

### 4.4. Limitations

The present study may have some limitations. First, we recognized that the lack of measurement data in the NHI database such as biochemical profiles, inflammatory biomarkers, gut microbiota and genetic factors could be a limitation. Therefore, we could only use disease diagnostic codes as surrogates for adjustment. We could not exclude the possibility of residual confounding from unmeasured confounders.

Second, we did not have clinical data or laboratory reports such as visual field test, optical coherence tomography and fluorescein angiography for AMD confirmation, subtype classification and severity assessment.

Third, misclassification of disease diagnoses in the database was possible. However, the misclassification should be nondifferential and the HRs might only be biased toward the null. The robustness of the finding of a preventive role of metformin in AMD could be supported by the consistency in different analyses (Table 1 and Table 2).

### 4.5. Strengths

The large population-based database and the careful design of the study provided several strengths. First, this study should be free from selection bias because the coverage rate of the NHI is high. A lack of statistical power was unlikely because the sample size was large, the enrollment period from 1999 to 2005 was long and the follow-up duration from 2006 to 2011 was also long. Therefore, we have more confidence to generalize the findings to the whole population.

Second, by using preexisting medical records, we have avoided recall bias and self-reporting bias.

Third, because only patients with a new diagnosis of diabetes mellitus were included and we defined metformin use since its initiation (Figure 2), the results were not distorted by prevalent user bias.

Fourth, immortal time bias might have resulted if we had inappropriately assigned the treatment status and/or we had miscalculated the follow-up time. In the present study, the possibility of enrolling non-diabetic people into the study was minimal because we restricted the enrollment of studied patients to those who had received prescription of antidiabetic drugs at least twice (Figure 2). Metformin treatment status was unlikely to be misclassified and we could more accurately calculate the cumulative duration and cumulative dose because we had the longitudinal information of drug prescription. Additionally, in the calculation of follow-up person-years, we deliberately excluded the following possibilities of immortal time: (1) the immortal time between diabetes diagnosis and the initiation of antidiabetic drugs and (2) the immortal time during the initial follow-up period of <18 months (Figure 2). It is worth pointing out that the immortal time between hospital discharge and the refill of discharged drugs would not happen in Taiwan because all discharge drugs can be obtained from the hospital on the same day of discharge.

Fifth, although different socioeconomic statuses might lead to a serious problem of detection bias in some countries, this is a relatively minor issue in Taiwan because the cost-sharing is very low in the NHI healthcare system. Actually, many medical expenses can be waived in patients who receive prescription refills for chronic diseases, in patients with low income and in veterans.

## 5. Conclusions

This study supports a preventive role of metformin in AMD development in a dose–response manner. However, the benefit of metformin on AMD is not significant in patients with diabetic retinopathy and such a benefit of metformin can only be demonstrated for nonexudative AMD. These findings should better be confirmed by the ongoing clinical trials because we could not exclude some inherent limitations associated with the observational study design of the study. As a result of the multiple benefits of metformin beyond glycemic control, including the prevention of AMD, it is deemed appropriate to recommend metformin as the first-line antidiabetic drug.

## Figures and Tables

**Table 1:** Incidence rates of age-related macular degeneration and hazard ratios with regard to metformin use.

Metformin Use	Incident Case Number	Cases Followed	Person-Years	Incidence Rate (Per 100,000 Person-Years)	Hazard Ratio	95% Confidence Interval	*p*-Value
Never users	639	13,303	62,855.47	1016.62	1.000		
Ever users	506	13,303	64,978.49	778.72	0.756	(0.673–0.850)	<0.0001
**Tertiles of cumulative duration of metformin therapy (months)**							
Never users	639	13,303	62,855.47	1016.62	1.000		
<31.8	191	4386	17,487.30	1092.22	1.131	(0.961–1.330)	0.1381
31.8–63.9	186	4388	21,823.66	852.29	0.821	(0.697–0.967)	0.0181
>63.9	129	4529	25,667.53	502.58	0.464	(0.384–0.561)	<0.0001
**Tertiles of cumulative dose of metformin therapy (grams)**							
Never users	639	13,303	62,855.47	1016.62	1.000		
<947.1	194	4389	17,679.78	1097.30	1.131	(0.962–1.329)	0.1352
947.1–2193.5	169	4390	21,997.90	768.26	0.739	(0.624–0.876)	0.0005
>2193.5	143	4524	25,300.82	565.20	0.525	(0.438–0.629)	<0.0001
**Tertiles of defined daily dose of metformin therapy per day**							
Never users	639	13,303	62,855.47	1016.62	1.000		
<0.49	158	4390	20,483.93	771.34	0.761	(0.640–0.906)	0.0021
0.49–0.64	184	4390	21,484.15	856.45	0.832	(0.706–0.980)	0.0274
>0.64	164	4523	23,010.41	712.72	0.684	(0.576–0.812)	<0.0001

**Table 2:** Sensitivity analyses comparing risk of age-related macular degeneration between ever users and never users of metformin in various subgroups of patients.

Model/Metformin Use	Incident Case Number	Cases Followed	Person-Years	Incidence Rate (Per 100,000 Person-Years)	Hazard Ratio	95% Confidence Interval	*p*-Value
**1. Analysis restricted to patients enrolled from 1999 to 2002**							
Never users	279	5979	27,844.59	1001.99	1.000		
Ever users	331	7984	39,501.77	837.94	0.815	(0.695–0.956)	0.0118
**2. Analysis restricted to patients enrolled from 2003 to 2005**							
Never users	360	7324	35,010.89	1028.25	1.000		
Ever users	175	5319	25,476.73	686.90	0.668	(0.558–0.801)	<0.0001
**3. Including only patients aged 50–64 years**							
Never users	246	6534	31,625.48	777.85	1.000		
Ever users	205	6512	32,740.69	626.13	0.794	(0.660–0.956)	0.0148
**4. Including patients aged 65–79 years**							
Never users	393	6769	31,229.99	1258.41	1.000		
Ever users	301	6791	32,237.80	933.69	0.733	(0.631–0.851)	<0.0001
**5. Including only male patients**							
Never users	330	7109	33,515.89	984.61	1.000		
Ever users	274	7212	35,057.37	781.58	0.784	(0.668–0.920)	0.0029
**6. Including only female patients**							
Never users	309	6194	29,339.59	1053.18	1.000		
Ever users	232	6091	29,921.12	775.37	0.727	(0.613–0.862)	0.0002
**7. Excluding patients with a diagnosis of anemia and/or nutritional deficiencies**							
Never users	472	9591	46,129.75	1023.20	1.000		
Ever users	376	10,088	49,569.05	758.54	0.736	(0.643–0.843)	<0.0001
**8. Patients with diabetic retinopathy**							
Never users	56	810	3862.97	1449.66	1.000		
Ever users	43	773	3768.50	1141.04	0.780	(0.524–1.160)	0.2199
**9. Patients without diabetic retinopathy**							
Never users	583	12,493	58,992.50	988.26	1.000		
Ever users	463	12,530	61,209.99	756.41	0.755	(0.668–0.853)	<0.0001
**10. Outcome defined as nonexudative age-related macular degeneration**							
Never users	495	13,303	63,198.84	783.24	1.000		
Ever users	361	13,303	65,244.76	553.30	0.697	(0.609–0.799)	<0.0001
**11. Outcome defined as exudative age-related macular degeneration**							
Never users	23	13,303	64,237.81	35.80	1.000		
Ever users	22	13,303	65,868.88	33.40	0.924	(0.515–1.658)	0.7919
**12. All covariates defined at censor**							
Never users	639	13,303	62,855.47	1016.62	1.000		
Ever users	506	13,303	64,978.49	778.72	0.756	(0.673–0.850)	<0.0001

**Table 3:** Characteristics of never and ever users of metformin matched on propensity score.

Variables	Never Users		Ever Users		Standardized Difference
	(*n* = 13,303)		(*n* = 13,303)		
	*n*	%	*n*	%	
Basic data					
Age (years) *	65.05	8.48	65.04	8.22	0.29
Sex (male)	7109	53.44	7212	54.21	1.47
Occupation					
I	4786	35.98	4749	35.70	
II	2321	17.45	2369	17.81	0.90
III	3345	25.14	3360	25.26	0.26
IV	2851	21.43	2825	21.24	−0.36
Living region					
Taipei	4450	33.45	4501	33.83	
Northern	1407	10.58	1370	10.30	−1.07
Central	2285	17.18	2372	17.83	1.66
Southern	2350	17.67	2294	17.24	−1.11
Kao-Ping and Eastern	2811	21.13	2766	20.79	−0.65
Major comorbidities commonly seen in diabetes patients					
Obesity	235	1.77	244	1.83	0.51
Hypertension	10,694	80.39	10,739	80.73	1.02
Dyslipidemia	8168	61.40	8069	60.66	−1.33
Complications related to diabetes					
Diabetic polyneuropathy	1373	10.32	1367	10.28	−0.33
Eye diseases	1091	8.20	995	7.48	−2.96
Nephropathy	3521	26.47	3556	26.73	0.16
Ischemic heart disease	5923	44.52	5963	44.82	0.59
Stroke	3981	29.93	4014	30.17	0.58
Peripheral arterial disease	2267	17.04	2289	17.21	0.31
Hypoglycemia	216	1.62	188	1.41	−1.89
Antidiabetic drugs					
Sulfonylurea	10,000	75.17	10,328	77.64	6.11
Acarbose	1461	10.98	1393	10.47	−2.93
Meglitinide	1106	8.31	1045	7.86	−1.69
Rosiglitazone	418	3.14	438	3.29	0.50
Pioglitazone	329	2.47	341	2.56	0.25
Insulin	776	5.83	659	4.95	−5.23
Drugs commonly used by diabetes patients or drugs that may affect the outcome					
Statins	5494	41.30	5526	41.54	0.64
Fibrates	3665	27.55	3643	27.38	−0.20
Calcium channel blockers	8315	62.50	8386	63.04	1.23
Angiotensin converting enzyme inhibitors/angiotensin receptor blockers	8723	65.57	8657	65.08	−0.95
Aspirin	7070	53.15	7074	53.18	0.23
Non-steroidal anti-inflammatory drugs **	5166	38.83	5163	38.81	0.00
Selective serotonin re-uptake inhibitors	1020	7.67	916	6.89	−3.03
Opioid analgesics	2100	15.79	2101	15.79	−0.12
Immunosuppressants **	687	5.16	656	4.93	−1.22
Common comorbidities that may affect the exposure/outcome					
Chronic obstructive pulmonary disease	5923	44.52	5842	43.91	−1.09
Tobacco abuse	176	1.32	164	1.23	−0.77
Alcohol-related diagnoses	578	4.34	534	4.01	−2.03
Head injury	140	1.05	162	1.22	1.32
Dementia	871	6.55	848	6.37	−0.67
Parkinson’s disease	383	2.88	407	3.06	0.96
Heart failure	2390	17.97	2323	17.46	−1.50
Valvular heart disease	1372	10.31	1308	9.83	−1.65
Gingival and periodontal diseases	9972	74.96	10,028	75.38	1.02
Pneumonia	1446	10.87	1415	10.64	−1.02
Osteoporosis	2915	21.91	2936	22.07	0.48
Arthropathies and related disorders	9591	72.10	9656	72.59	1.30
Psoriasis and similar disorders	274	2.06	307	2.31	1.72
Dorsopathies	9344	70.24	9348	70.27	0.12
Liver cirrhosis	600	4.51	559	4.20	−1.86
Hepatitis B virus infection	180	1.35	126	0.95	−4.24
Hepatitis C virus infection	567	4.26	541	4.07	−1.16
Other chronic non-alcoholic liver diseases	971	7.30	965	7.25	0.06
Organ transplantation	65	0.49	46	0.35	−2.63
Human immunodeficiency virus infection	6	0.05	4	0.03	−0.76
*Helicobacter pylori* infection	70	0.53	76	0.57	0.73
Peptic ulcer site unspecified	5067	38.09	4997	37.56	−1.04
Appendicitis	198	1.49	204	1.53	0.37
Irritable bowel syndrome	1728	12.99	1637	12.31	−2.12
Noninfective enteritis and colitis	5990	45.03	5919	44.49	−1.02
Abscess of anal/rectal regions	143	1.07	142	1.07	−0.24
Anal fissure/fistula	265	1.99	262	1.97	−0.09
Episodic mood disorders	642	4.83	565	4.25	−2.79
Depressive disorder	362	2.72	383	2.88	0.97
Suicidal attempt	4	0.03	3	0.02	−0.43
Insomnia	3126	23.50	3099	23.30	−0.45
Drug dependence	56	0.42	55	0.41	0.00
Diseases of the ear and mastoid process	5842	43.91	5901	44.36	1.00
Hearing loss	855	6.43	903	6.79	1.64
Inflammatory diseases of the central nervous system (encephalitis and meningitis)	149	1.12	156	1.17	0.42
Tuberculosis	443	3.33	436	3.28	−0.38
Malaria	4	0.03	1	0.01	−2.36
Some parasitic diseases	900	6.77	834	6.27	−1.98
Epilepsy and recurrent seizures	316	2.38	270	2.03	−2.55
Disorders of fluid electrolyte and acid-base balance	1218	9.16	1150	8.64	−2.15
Cancer during follow-up	1485	11.16	1463	11.00	−0.61

The different classes of occupation are depicted in “Materials and Methods”. * Age is expressed as mean and standard deviation. ** Defined as a continuous use of ≥90 days.

## Data Availability

The datasets presented in this article are not readily available because public availability of the dataset is restricted by local regulations to protect privacy. Requests to access the datasets should be directed to the corresponding author, ccktsh@ms6.hinet.net.

## Supplementary Materials

The following supporting information can be downloaded at: https://www.mdpi.com/article/10.3390/ph16020224/s1, Table S1: Incidence of age-related macular degeneration by age and sex in patients with type 2 diabetes mellitus by including patients of all ages in the unmatched cohort.
