# Supplementary material for: The Risk of Age-Related Macular Degeneration Is Reduced in Type 2 Diabetes Patients Who Use Metformin
Source: Pharmaceuticals (Basel). 2023 Feb 1;16(2):224. doi: 10.3390/ph16020224 (PMC9963185; doi:10.3390/ph16020224)
Supplement: Supplementary file 1 [file pharmaceuticals-16-00224-s001.zip › pharmaceuticals-2051545-supplementary.pdf]

Supplementary Table S1. Incidence of age-related macular degeneration by age and sex in patients with type 2 diabetes mellitus by including patients of all ages in the unmatched cohort.

	Men					Women					Both sexes				
Age group (years)	Incident case number	Cases followed	%	Person-years	Incidence rate (per 100,000 person-years)	Incident case number	Cases followed	%	Person-years	Incidence rate (per 100,000 person-years)	Incident case number	Cases followed	%	Person-years	Incidence rate (per 100,000 person-years)
<50	622	46921	1.33	236373.95	263.14	406	27035	1.50	137545.88	295.17	1028	73956	1.39	373919.83	274.93
50-54	614	27825	2.21	137906.46	445.23	505	21243	2.38	108522.26	465.34	1119	49068	2.28	246428.72	454.09
55-59	770	26440	2.91	128256.97	600.36	776	23627	3.28	117585.75	659.94	1546	50067	3.09	245842.71	628.86
60-64	728	19094	3.81	92298.25	788.75	861	19769	4.36	97351.01	884.43	1589	38863	4.09	189649.26	837.86
65-69	753	16632	4.53	79261.57	950.02	982	19691	4.99	95442.07	1028.90	1735	36323	4.78	174703.64	993.11
70-74	625	12357	5.06	57547.90	1086.05	751	15644	4.80	73914.41	1016.04	1376	28001	4.91	131462.31	1046.69
75-79	463	8941	5.18	40225.41	1151.01	469	10149	4.62	45879.39	1022.25	932	19090	4.88	86104.80	1082.40
80-84	176	3934	4.47	16425.63	1071.50	187	4922	3.80	20720.42	902.49	363	8856	4.10	37146.05	977.22
85+	48	1224	3.92	4681.17	1025.38	57	1938	2.94	7375.85	772.79	105	3162	3.32	12057.02	870.86
Total	4799	163368	2.94	792977.32	605.19	4994	144018	3.47	704337.03	709.04	9793	307386	3.19	1497314.35	654.04
